# Long-Term Clinical Outcomes of Unprotected Left Main Percutaneous Coronary Intervention: A Large Single-Centre Experience

**DOI:** 10.1155/2021/8829686

**Published:** 2021-01-12

**Authors:** Lijian Gao, Zhan Gao, Ying Song, Changdong Guan, Bo Xu, Jue Chen, Haibo Liu, Xuewen Qin, Min Yao, Jinqing Yuan, Yongjian Wu, Fenghuan Hu, Jie Qian, Yida Tang, Kefei Dou, Weixian Yang, Hong Qiu, Chaowei Mu, Jun Dai, Shubin Qiao, Jilin Chen, Runlin Gao, Yuejin Yang

**Affiliations:** ^1^Department of Cardiology, Coronary Heart Disease Center, Fuwai Hospital, National Center for Cardiovascular Diseases, Chinese Academy of Medical Science and Peking Union Medical College, Beijing, China; ^2^Catheterization Laboratories, Fu Wai Hospital, National Center for Cardiovascular Diseases, Chinese Academy of Medical Sciences, Beijing, China

## Abstract

**Aims:**

This study sought to report the 10-year clinical outcomes of patients who underwent unprotected left main (LM) percutaneous coronary intervention (PCI) in a large centre.

**Methods and Results:**

A total of 913 consecutive patients who underwent unprotected LM PCI from January 2004 to December 2008 at Fu Wai Hospital were retrospectively analysed; the mean age was 60.0 ± 10.9 years, females accounted for 22% of patients, diabetes was present in 27.7% of patients, and an LM bifurcation lesion occurred in 82.9% of patients. During the median follow-up of 9.7 years, major adverse cardiac or cerebrovascular events (MACCEs) occurred in 25.6% (234) of patients, and the rates of all-cause death, myocardial infarction, and stroke were 14.9%, 11.0%, and 7.1%, respectively. Cardiac death occurred in only 7.9% of patients. The estimated event rate was 41.9% for death/myocardial infarction/any revascularization and 45.9% for death/MI/stroke/any revascularization. Definite/probable stent thrombosis occurred in 4.3% (39) of patients. According to the subgroup analysis, IVUS-guided PCI was associated with less long-term MACCEs. Further multivariate analysis identified that age and LVEF<40% were the only independent predictors for 10-year death. Age, LVEF<40%, creatinine clearance, and incomplete revascularization were independent predictors for death/MI, while a two-stent strategy, diabetes, a transradial approach, and the use of bare metal stents (BMSs) or first-generation drug-eluting stents (DESs) were not.

**Conclusions:**

Unprotected LM PCI in a large cohort of consecutive patients in a single large centre demonstrated favourable long-term outcomes up to 10 years even with the use of BMSs and first-generation of DESs.

## 1. Introduction

Although randomized controlled trials comparing the effect of percutaneous coronary interventions (PCIs) and coronary artery bypass graft (CABG) still have not reached consistent results in patients with unprotected left main (LM) coronary artery disease (CAD) [1, 2], PCI has always been recommended as an effective treatment for patients with unprotected LM CAD by guidelines [3, 4] and has been performed in daily practice, except in bifurcated lesion and the two-stent strategy [5].

During the last 2 decades, therapeutic advancements including drug-eluting stents (DESs) [6–11] and invasive imaging tools such as intravascular ultrasound (IVUS) [12–17] have largely improved PCI outcomes in patients with unprotected LM disease. In addition, increased experience in complex LM stenting [18] has further improved interventional device-oriented outcomes [19]. However, the long-term outcomes of patients undergoing LM PCI have always been a concern due to suboptimal results, such as stent underexpansion or malapposition at this particular part, which make patients prone to stent thrombosis and in-stent restenosis, leading to devastating consequences, including death [20–22]. Previous reports have revealed more favourable outcomes in LM patients who underwent PCI than in those who underwent CABG or medical therapy alone; however, the long-term follow-up results showed conflicting reversed findings [23, 24]. On the other hand, clinical or technique factors that influence long-term outcomes following LM are still controversial. In this circumstance, we retrospectively collected over 900 consecutive LM patients with detailed patient demographics, lesion, and procedural information who underwent LM PCI at a large cardiac centre with as long as a 10-year follow-up duration. The study sought to analyse the very long-term performance of PCI for LM disease and investigate potential factors that influence long-term outcomes.

## 2. Methods

### 2.1. Study Population

**Figure 1:**
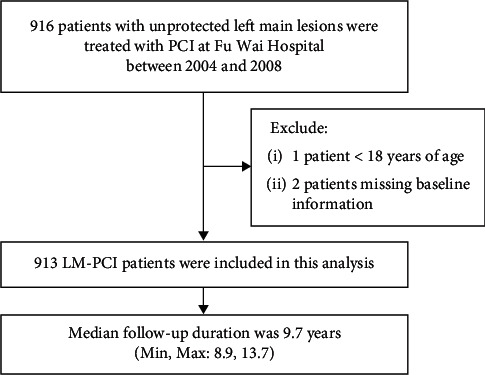
Patient flow. PCI = percutaneous coronary intervention; LM = left main.

Between January 2004 and December 2008, 19,600 patients underwent PCI at Fu Wai Hospital; among them, a total of 916 consecutive patients diagnosed with LM diseases were retrospectively collected. The exclusion criteria were age <18 years and missing major baseline information. After excluding 3 patients who met the exclusion criteria, 913 LM PCI patients were finally analysed (Figure 1). Unprotected LM disease was defined as documented myocardial ischaemia with ≥50% LM stenosis and no patent bypass graft to the left anterior descending or left circumflex arteries. The population of patients who were rejected by surgeons from surgery included those who met any 1 of the following criteria: chronic obstructive pulmonary disease, left ventricular ejection fraction <35% with minimal or without viable myocardium, age >70 years, acute myocardial infarction with haemodynamic instability, creatinine clearance <50 ml/min, or bleeding history within 6 months. Clinical, procedural, and outcomes data were recorded in a dedicated database. The baseline and residual SYNTAX Score (SS) were assessed using standard quantitative coronary analysis methodology by an independent angiographic core laboratory. Follow-up was performed via an office visit or telephone contact at 30 days and annually thereafter.

### 2.2. Procedures

All patients undergoing PCI were prescribed aspirin plus clopidogrel (loading dose, 300 or 600 mg) before the coronary intervention unless they had previously received regular antiplatelet medications. Procedures were performed with standard interventional techniques. Lesions in the ostium or body of the LM usually received a single stent with the postdilation technique; if the single stent crossed over the LM bifurcation to the LAD, postdilatation with kissing balloon angioplasty was used at the operator's discretion to finish the procedure. When treating distal LM bifurcation, the operators decided the strategy of a 1- or 2-stent technique. When the 2-stent strategy was applied, the proximal optimization technique and postdilatation with kissing balloon angioplasty was mandatory to achieve complete apposition of the LM stent. The use of intravascular ultrasound (IVUS) or optical coherence tomography (OCT) was at the operator's discretion, and an intra-aortic balloon bump (IABP) was used as mechanical support in patients with a very low LVEF or other complications. The use of dual antiplatelet therapy with aspirin and clopidogrel was recommended for, at least, 12 months after stent implantation.

### 2.3. Endpoints and Definitions

The present study evaluated the long-term safety and efficacy following LM PCI. The primary safety endpoint was the composite endpoint of death, MI, and stroke. The primary efficacy endpoint was target-vessel revascularization (TVR). The secondary endpoints included individual components of the composite outcome, cardiac death, any revascularization, target lesion revascularization (TLR), and stent thrombosis as defined according to definite or probable Academic Research Consortium (ARC) criteria [22]. Cardiac death was defined as any death that could not be attributed to a noncardiac cause. Periprocedural MI was defined as a creatine kinase concentration >2 times the upper limit of normal within 48 hours after the procedure, and TVR was defined as any revascularization within the entire major coronary vessels proximal or distal to a target lesion, including upstream and downstream side branches and the target lesion itself.

### 2.4. Statistical Analysis

Continuous variables are presented as the mean ± SD and were compared by Student's t-test. Categorical variables are presented as percentages and counts; between-group differences were compared by the chi-square test or Fisher's exact test. Subgroup analyses were performed to identify long-term outcome predictors after LM PCI among different populations including patients who were suitable for surgery versus the regular population, the transradial versus the transfemoral approach, bifurcation versus nonbifurcation lesions, one- versus two-stent strategy, BMS versus DES treatments, and treatment with or without IVUS guidance. The ten-year outcomes in the overall population and subgroups are presented as Kaplan–Meier estimates and were compared using the log-rank test. Multivariable Cox proportional hazards models were constructed to identify independent predictors of 10-year all-cause death, cardiac death, and death/MI. All statistical analyses were performed using SAS version 9.1.3 (SAS Institute, Cary, North Carolina).

## 3. Results

### 3.1. Clinical and Procedural Characteristics

Baseline characteristics are presented in Table 1. A total of 913 LM PCI patients with the mean age of 60.0 ± 10.9 years were enrolled; females accounted for 22.0% of patients, diabetes was present in 27.7% of patients, and unstable angina was present in 60.4% of patients. An LM bifurcation lesion was present in 82.9% of patients, LM plus 3-vessel disease was present in 36.1% of patients, and the mean LVEF was 62.5 ± 7.9%. Patients with a SYNTAX score ≤32 and >32 accounted for 86.3% and 13.7%, respectively. LM PCI was performed with a transradial approach in 45.7% of the patients, the 2-stent strategy in 26.5%, and IVUS guidance in 39.5%, as shown in Table 2.

**Figure 2:**
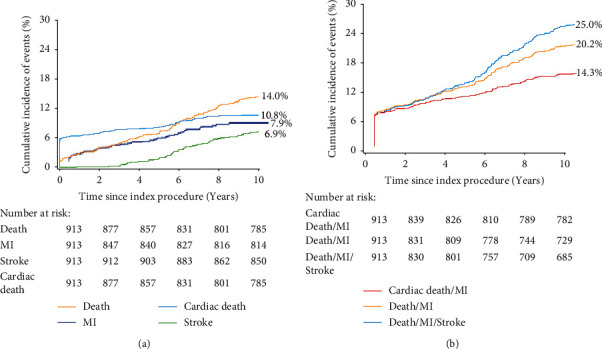
Long-term efficiency and safety after LM PCI. (a) Kaplan–Meier curves for death, cardiac death, MI, and stroke events; (b) Kaplan–Meier curves for composite events including death/MI and death/MI/stroke. MI = myocardial infarction.

**Figure 3:**
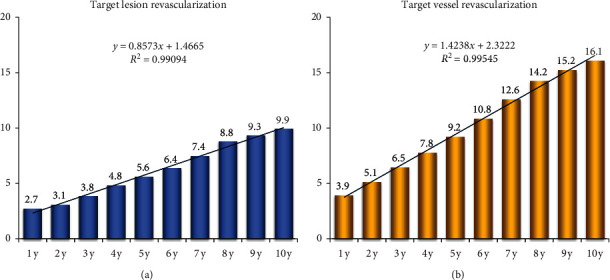
10-year target lesion revascularization and target-vessel revascularization. Annual target lesion revascularization (a) and target-vessel revascularization (b) event rates.

The median follow-up duration of these patients was 9.7 years (min, max: 8.9, 13.7 years). The composite endpoint of death/MI/stroke occurred in 234 (25.6%, 95% confidence interval [CI]: 22.7–28.5%) patients, the rate of all-cause death was 14.9% (136), and 11.0% (100) and 7.1% (65) of patients suffered MI and stroke, respectively (Table 3 and Figure 2). The 10-year estimated incidence of any revascularization was 25.0%, and the TVR and target lesion revascularization (TLR) were 16.1% and 9.9%, respectively, with increase rates 1.4% (TVR) and 0.9% (TLR) annually (Figure 3). Up to 10 years, 136 (14.9%) patients died; among them, 72 (7.9%) patients died due to cardiac events (Appendix). Definite/probable stent thrombosis occurred in 39 (4.3%) patients.

### 3.2. Subgroup Analyses

**Figure 4:**
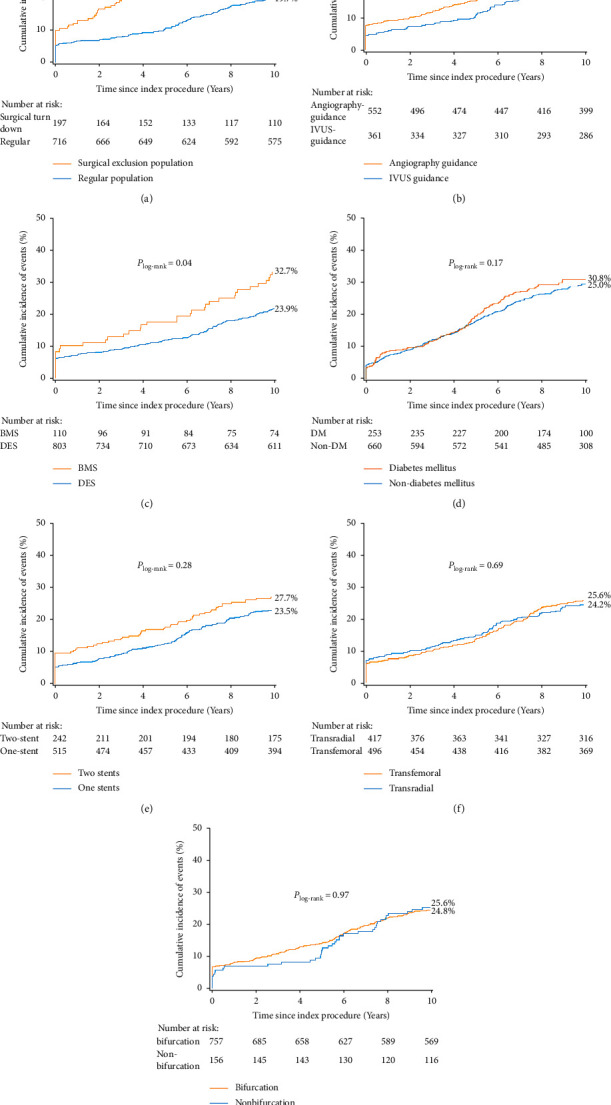
Survival curves of 10-year death/MI/stroke events among subgroups. The surgical exclusion population included patients meeting any one of the following criteria: chronic obstructive pulmonary disease, left ventricular ejection fraction <35%, age >70, acute myocardial infarction with haemodynamic instability, creatinine clearance <50, or bleeding history within 6 months. BMS = bare metal stent; DES = drug-eluting stent; and IVUS = intravascular ultrasound. Other abbreviations are as in Figure 2.

A total of 197 (21.6%) patients in the present study were rejected by surgeons due to patient comorbidities or surgical ineligibility. Kaplan–Meier curves showed that there were no differences between patients who underwent the transradial or transfemoral approach (log-rank *p*=0.69), those with LM bifurcation or nonbifurcation lesions (log-rank *p*=0.97), or those treated with the two-stent or one-stent strategy (log-rank *p*=0.28), while the 10-year death/MI/stroke rate was significantly higher in patients who were rejected by surgeons (log-rank *p* < 0.0001) and in patients with implantation of BMS (32.7% vs. 23.9%, log-rank *p*=0.04). On the other hand, an LM PCI procedure guided by IVUS significantly reduced long-term death/MI/stroke (20.8% vs. 27.7%, log-rank *p*=0.03) (Figure 4).

### 3.3. Multivariate Analysis

In the multivariate analysis, we found that age and a left ventricular eject fraction (LVEF) < 40% were independent predictors for 10-year death (all *p* < 0.01). In addition, age, LVEF<40%, creatinine clearance, and incomplete revascularization were independent predictors for 10-year death/MI (all *p* < 0.01) (Table 4).

## 4. Discussion

In this study, the long-term outcomes of patients after unprotected LM PCI were assessed in a large cohort of real-world patients in a large Chinese cardiovascular centre. The major findings of this study were as follows: (1) even with the use of a BMS or first-generation DES, PCI for unprotected LM disease showed favourable long-term results for up to 10 years; (2) compared with BMSs, DESs significantly reduced long-term adverse events, and IVUS-guided PCI was associated with a lower incidence of the composite death, stroke, or MI events; and (3) age and an LVEF < 40% are independent predictors for 10-year death, while age, LVEF < 40%, creatinine clearance, and incomplete revascularization are independent predictors for 10-year death/MI.

LM PCI outcomes after a 10-year follow-up duration are scarcely reported. Patients in the MAINCOMPARE (Revascularization for Unprotected Left Main Coronary Artery Stenosis: Comparison of Percutaneous Coronary Angioplasty Versus Surgical Revascularization) registry [25] who received BMSs for LM with less complex CAD showed a 10-year survival probability of 83.1%. In the LE MANS (Left Main Coronary Artery Stenting) registry, which included a wide spectrum of patients with CAD, as well as acute coronary syndromes, the 10-year survival after LM stenting was nearly 70% [11]. In the LE MANS prospective trial, which randomly evaluated LM stenting and CABG for unprotected LM stenosis with low and medium SYNTAX scores, the patients in the PCI arm reached nearly 80% 10-year survival [26]. In the ASAN-MAIN (ASAN Medical Center-Left MAIN Revascularization) registry, the 10-year survival was 84.1% in patients with LM bare metal stenting [27]. In a recent report comparing provisional stenting vs. the two-stent strategy in patients with LM bifurcation lesions, the 10-year survival of the overall patients was over 70% [28]. To our knowledge, this study included the largest cohort of 913 LM PCI patients with 10-year follow-up results. In this study, the 10-year estimated rate of all-cause death was 14.9% with cardiac death accounting for only 7.9%, which was even lower than the results of the abovementioned studies. Overall, the results of this study together with those of the others mentioned above suggest that the very long-term outcomes of PCI for LM were acceptable. Furthermore, a recent study demonstrated that LM PCI using DESs in those patients with high-risk features that represent exclusion criteria of previous randomized trials (e.g., AMI within 1 week, LVEF < 30%, and cardiogenic shock) achieved the same long-term outcomes compared with low-risk patients [29]. Our latest study analysed all PCI patients in 2013 in our centre, and during a 2-year follow-up period, we found that LM PCI was not an independent risk factor for any clinical adverse events [30]. Thus, it seems that interventionists should not consider LM lesions per se as a particular high-risk subgroup any more with contemporary PCI treatment. On the other hand, in this retrospective study, a large portion of the patients (21.6%) evaluated for LM PCI was rejected by surgeons due to patient comorbidities or surgical ineligibility, and those patients were proven to have a worse long-term prognosis.

After the introduction of DESs, with a remarkable reduction in restenosis and repeat revascularization, LM PCI with DESs has been confirmed to have more favourable clinical outcomes than that with BMSs [6–11]. However, it could be associated with increased risk of very late stent thrombosis. Due to inaccuracy of angiography in LM coronary stenosis assessment [31], IVUS guidance has been proven to be more important than for non-LM lesions and improve the long-term prognosis in patients with unprotected LM CAD undergoing PCI [18, 32–34]. Our study had same findings: compared with BMSs, first-generation DESs significantly reduced long-term adverse events. The majority of definite/probable stent thrombosis was very late thrombosis, and it may be partly explained by the predominant use of DESs in this population; IVUS-guided PCI was associated with a lower incidence of the composite death, stroke, or MI events. This retrospective study revealed no difference in the long-term prognosis with the 1- or 2-stent strategy. Most patients with LM bifurcations received the 1-stent strategy, while the 2-stent strategy was mainly chosen for LM bifurcation lesions with more complex anatomy or true bifurcations and was performed by high-volume operators [19]. The techniques used in this series were similar to European Bifurcation Club recommendations [32]. In this observational study, DM status was not seen to be significantly associated with worse long-term adverse events, and this finding was inconsistent with previous reports [35–38].

This study demonstrated that age and an LVEF < 40% were independent predictors of 10-year death. On the other hand, age, LVEF<40%, creatinine clearance, and incomplete revascularization were independent predictors for 10-year death and MI. This finding was consistent with our previous short-term (15-month follow-up) small cohort (220 LM PCI patients) study from 2003 to 2006 in our centre, which also found that an LVEF < 40% and incomplete revascularization (residual SS ≥ 8) were independent predictors for death and MI after multivariate analysis [39]. These results were also consistent with two recently published long-term studies [40, 41].

Our study has potential clinical implications. It seems that interventionists should be more optimistic for the very long-term outcomes of LM PCI, but there still are some issues that need to be noted. First, these consecutive 916 LM PCI cases from 2004 to 2008 only accounted for 4.67% of the total PCI cases at the same time; a large proportion of patients with LM disease were recommended to undergo CABG treatment. Second, just as our previous study demonstrated that operator experience affected prognosis after LM PCI [17], whether the conclusions of this study achieved in a large cardiac centre where most LM PCIs were performed by experienced operators [19] could be expanded to other centres needs to be further confirmed.

## 5. Study Limitations

This report has some limitations that should be acknowledged. First, the major limitation of this study is its observational design, which introduces latent, unrecognized, or unmeasured variables that could result in hidden bias. Second, this study only included patients who underwent LM PCI; therefore, the very long-term outcomes of LM PCI in comparison with those of LM-CABG cannot be evaluated by this study. Third, the study used a creatine kinase concentration >2 times to define periprocedural MI, which is an old definition that might overestimate MI rates. Finally, these data are from 2004 to 2008, which cannot reveal the latest advances in the PCI era. Including patients with now-historical stents, this report does not include physiologic assessment (FFR/iFR, etc) and has limited use of imaging. However, with utility of these modern assessment modalities, one would expect even more favourable results of PCI for LM disease in contemporary practice.

## 6. Conclusions

The current report, drawn from a large cohort of consecutive patients who underwent LM PCI, indicated that even with the use of BMSs or first-generation DESs, PCI for unprotected LM disease showed favourable long-term results for up to 10 years. In addition, age and the LVEF are key factors for long-term prognosis following LM PCI. Further study should focus on the long-term outcomes of LM PCI in comparison with LM-CABG to provide more evidence.

## Figures and Tables

**Table 1:** Baseline patient characteristics.

	*N* = 913
Age, years	60.0 ± 10.9
Female	22.0% (201)
Body mass index, kg/m^2^	25.6 ± 3.1
Diabetes	27.7% (253)
Insulin requiring	0.4% (4)
Current smoking	33.4% (305)
Hypertension	59.6% (544)
Hyperlipidaemia	49.8% (455)
Family history of coronary artery disease	14.5% (132)
Previous percutaneous coronary intervention	24.1% (220)
Prior myocardial infarction	30.9% (282)
Prior stroke	6.9% (63)
Peripheral arterial disease	3.3% (30)
Unstable angina	60.4% (551)
LVEF, %	62.5 ± 7.9
LVEF<40%	1.3% (12)
LVEF 40%–50%	5.8% (53)
LVEF > 50%	92.9% (848)

Values are reported as the mean ± SD or % (*n*). LVEF = left ventricular ejection fraction.

**Table 2:** Baseline lesion and procedure characteristics.

	*N* = 913
*Coronary artery disease extent*	
Isolated LM	8.0% (73)
LM + 1VD	19.9% (182)
LM + 2VD	35.9% (328)
LM + 3VD	36.1% (330)
Total occluded lesion	4.4% (40)

*LM lesion location*	
Ostium	11.4% (104)
Shaft	5.7% (52)
Bifurcation	82.9% (757)

*Main vessel*	
Reference vessel diameter, mm	3.66 ± 0.46
Lesion length, mm	20.7 ± 15.3
Diameter stenosis, %	83.0 ± 10.7

*Side branch*	
Reference vessel diameter, mm	2.96 ± 0.38
Lesion length, mm	20.3 ± 13.7
Diameter stenosis, %	79.8 ± 13.2

*Medina type for bifurcation lesion*	
0,1,1	4.1% (37)
1,0,0	3.8% (29)
1,0,1	7.9% (60)
1,1,0	46.8% (354)
1,1,1	36.6% (277)

*Lesion type*	
De novo	96.4% (880)

*SYNTAX score*	24.5 ± 7.4
SYNTAX score ≤ 32	86.3% (788)
SYNTAX score > 32	13.7% (125)

*Procedure access*	
Transradial approach	45.7% (417)
Transfemoral approach	54.3% (496)

*Stent type*	
Bare metal stent	12.0% (110)
Drug-eluting stent	88.0% (803)
1st generation drug-eluting stent	15.0% (137)
2nd generation drug-eluting stent	72.9% (666)
Number of stents per patient	2.15 ± 1.18
Total stent length, mm	32.1 ± 19.3
2-stent strategy	26.5% (242)
Crush	16.3% (149)
T-stent	3.3% (30)
V or kissing stent	5.4% (49)
Culotte	1.5% (14)
Residual SYNTAX score	4.86 ± 6.26
Guidance with IVUS	39.5% (361)
Performed by an experienced operator*∗*	83.2% (760)

*∗*Experienced operator defined as those performed, at least, 15 LM PCIs per year for, at least, 3 consecutive years.

**Table 3:** 10-year clinical outcomes.

	Estimated event rates *N* = 913	95% confidence interval
All-cause death	14.9% (136)	12.5%–17.3%
Cardiac death	7.9% (72)	6.1%–9.7%
MI	11.0% (100)	8.9%–13.0%
Periprocedural MI*∗*	3.3% (30/913)	3.0%–3.6%
Target-vessel-related MI	9.7% (89)	7.8%–11.7%
Stroke	7.1% (65)	5.4%–8.8%
Any revascularization	25.0% (228)	22.1%–27.8%
TVR	16.1% (147)	13.7%–18.5%
TLR	9.9% (90)	7.9%–11.8%
Definite/probable ST	4.3% (39)	2.9%–5.6%
Definite ST	1.2% (11)	0.4%–2.0%
Probable ST	3.1% (28)	1.9%–4.2%
Acute	0.2% (2)	0%–0.6%
Subacute	0.4% (4)	0%–0.9%
Late	0.3% (3)	0%–0.8%
Very late	3.3% (30)	2.1%–4.5%
Death + stroke + MI	25.6% (234)	22.7%–28.5%
Cardiac death + target-vessel MI + TLR	23.1% (211)	20.3%–25.9%
Death + MI + any revascularization	41.9% (383)	38.7%–45.2%
Death + MI + stroke + any revascularization	45.9% (419)	42.6%–49.2%

Values are reported as % (*n*); *∗*periprocedural MI was defined as a creatine kinase concentration > 2 times the upper limit of normal within 48 hours after the procedure; TVR = target-vessel revascularization; TLR = target lesion revascularization; ST = stent thrombosis; and MI = myocardial infarction.

**Table 4:** Predictors of long-term adverse events after LM PCI.

	Hazard ratio (95% confidence interval)	*p* value
*Death*		
Bifurcation lesion	1.11 (0.70, 1.77)	0.66
EF<40%	4.51 (1.98, 10.28)	<0.001
Incomplete revascularization	1.06 (0.73, 1.55)	0.76
Diabetes	0.98 (0.67, 1.44)	0.92
Age	1.71 (1.37, 2.12)	<0.001
CCr	0.10 (0.92, 1.07)	0.89

*Death/MI*		
Bifurcation lesion	0.87 (0.73, 1.04)	0.66
EF<40%	1.89 (1.09, 3.28)	0.02
Incomplete revascularization	1.16 (1.01, 1.33)	0.03
Diabetes	1.05 (0.98, 1.22)	0.49
Age	1..15 (1.07, 1.24)	<0.001
CCr	1.04 (1.02, 1.06)	0.001

Incomplete revascularization was defined as a SYNTAX revascularization index < 100%. Abbreviations are as in Tables 1 and 2.

## Data Availability

The clinical and procedural data used to support the findings of this study are included within the article.

## Supplementary Materials

Supplementary Materials The supplementary file includes the appendix of table with detailed description of all-cause death
