# Supplementary material for: Long-Term Clinical Outcomes of Unprotected Left Main Percutaneous Coronary Intervention: A Large Single-Centre Experience
Source: J Interv Cardiol. 2021 Jan 12;2021:8829686. doi: 10.1155/2021/8829686 (PMC7815387; doi:10.1155/2021/8829686)
Supplement: Supplementary Materials — The supplementary file includes the appendix of table with detailed description of all-cause death [file 8829686.f1.docx]

**Appendix**

**Table. Detailed Description of all Cause Death**

| ID | PCI date | Cardiac Death | Detailed Description of Death |
| --- | --- | --- | --- |
| 1 | 2004-10-22 | Y | Uncertain |
| 2 | 2004-10-22 | N | Myocardial infarction |
| 3 | 2004-10-29 | Y | Myocardial infarction |
| 4 | 2004-12-16 | N | Sudden death |
| 5 | 2004-12-17 | N | Other reason |
| 6 | 2004-12-3 | N | Other reason |
| 7 | 2004-12-7 | N | Other reason |
| 8 | 2004-8-5 | Y | Uncertain |
| 9 | 2004-9-5 | Y | Uncertain |
| 10 | 2004-9-9 | Y | Sudden death |
| 11 | 2005-10-24 | N | Other reason |
| 12 | 2005-10-24 | N | Other reason |
| 13 | 2005-11-10 | Y | Uncertain |
| 14 | 2005-11-16 | N | Other reason |
| 15 | 2005-11-4 | N | Other reason |
| 16 | 2005-1-20 | Y | Uncertain |
| 17 | 2005-12-2 | Y | Uncertain |
| 18 | 2005-12-27 | N | Other reason |
| 19 | 2005-2-11 | Y | Uncertain |
| 20 | 2005-3-10 | N | Other reason |
| 21 | 2005-3-22 | N | Lung cancer |
| 22 | 2005-4-1 | N | Other reason |
| 23 | 2005-4-1 | N | Myeloma |
| 24 | 2005-4-15 | N | myeloma |
| 25 | 2005-4-15 | Y | Myocardial infarction |
| 26 | 2005-4-29 | Y | Uncertain |
| 27 | 2005-4-5 | N | Other reason |
| 28 | 2005-4-6 | Y | Uncertain |
| 29 | 2005-4-6 | Y | Uncertain |
| 30 | 2005-4-7 | Y | Sudden death |
| 31 | 2005-4-8 | N | Multiple organ dysfunction syndrome |
| 32 | 2005-5-10 | N | Other reason |
| 33 | 2005-5-11 | N | Stroke |
| 34 | 2005-5-19 | Y | Myocardial infarction |
| 35 | 2005-6-16 | N | Other reason |
| 36 | 2005-6-17 | Y | Myocardial infarction |
| 37 | 2005-6-30 | N | Other reason |
| 38 | 2005-7-22 | N | Stroke |
| 39 | 2005-7-4 | Y | Uncertain |
| 40 | 2005-8-31 | N | Other reason |
| 41 | 2005-8-4 | N | An accident |
| 42 | 2005-8-5 | Y | Sudden death |
| 43 | 2005-9-15 | N | Renal failure |
| 44 | 2005-9-22 | Y | Myocardial infarction |
| 45 | 2005-9-22 | Y | Uncertain |
| 46 | 2005-9-26 | N | Other reason |
| 47 | 2005-9-7 | Y | Sudden death |
| 48 | 2006-10-18 | Y | Uncertain |
| 49 | 2006-1-17 | N | Pancreatic cancer |
| 50 | 2006-11-9 | Y | Uncertain |
| 51 | 2006-12-15 | Y | Uncertain |
| 52 | 2006-12-21 | N | Other reason |
| 53 | 2006-12-21 | Y | Sudden death |
| 54 | 2006-1-24 | N | Other reason |
| 55 | 2006-12-5 | Y | Uncertain |
| 56 | 2006-12-6 | Y | Sudden death |
| 57 | 2006-12-7 | N | Other reason |
| 58 | 2006-1-9 | Y | Myocardial infarction |
| 59 | 2006-2-13 | N | Cancer-UK |
| 60 | 2006-2-16 | Y | Sudden death |
| 61 | 2006-2-22 | N | Other reason |
| 62 | 2006-2-22 | Y | Sudden death |
| 63 | 2006-3-3 | Y | Sudden death |
| 64 | 2006-4-29 | Y | Myocardial infarction |
| 65 | 2006-4-29 | Y | Uncertain |
| 66 | 2006-4-8 | N | Multiple organ dysfunction syndrome |
| 67 | 2006-5-11 | Y | Uncertain |
| 68 | 2006-5-22 | N | Other reason |
| 69 | 2006-5-9 | N | Other reason |
| 70 | 2006-6-4 | Y | Myocardial infarction |
| 71 | 2006-6-6 | Y | Myocardial infarction |
| 72 | 2006-7-27 | N | Other reason |
| 73 | 2006-8-10 | Y | Uncertain |
| 74 | 2006-8-10 | Y | Sudden death |
| 75 | 2006-8-19 | Y | Uncertain |
| 76 | 2006-8-23 | N | Other reason |
| 77 | 2006-8-25 | Y | Uncertain |
| 78 | 2006-8-28 | Y | Uncertain |
| 79 | 2006-8-4 | N | Other reason |
| 80 | 2006-9-11 | Y | Heart failure |
| 81 | 2006-9-21 | Y | Uncertain |
| 82 | 2007-10-17 | N | Myocardial infarction |
| 83 | 2007-10-18 | N | Multiple organ dysfunction syndrome |
| 84 | 2007-11-19 | Y | Uncertain |
| 85 | 2007-11-2 | N | Other reason |
| 86 | 2007-11-23 | N | Other reason |
| 87 | 2007-11-8 | N | Hematological diseases |
| 88 | 2007-12-19 | Y | Uncertain |
| 89 | 2007-1-22 | Y | Myocardial infarction |
| 90 | 2007-12-20 | N | Stroke |
| 91 | 2007-1-5 | Y | Uncertain |
| 92 | 2007-2-2 | Y | Myocardial infarction |
| 93 | 2007-3-28 | Y | Pulmonary embolism |
| 94 | 2007-3-6 | N | An accident |
| 95 | 2007-4-19 | Y | Uncertain |
| 96 | 2007-4-24 | Y | Myocardial infarction |
| 97 | 2007-6-19 | N | An accident |
| 98 | 2007-6-21 | Y | Heart failure |
| 99 | 2007-6-22 | N | Other reason |
| 100 | 2007-6-27 | Y | Sudden death |
| 101 | 2007-7-12 | Y | Uncertain |
| 102 | 2007-7-12 | N | Other reason |
| 103 | 2007-7-12 | N | Other reason |
| 104 | 2007-8-28 | Y | Sudden death |
| 105 | 2007-8-6 | N | Other reason |
| 106 | 2007-9-4 | N | Other reason |
| 107 | 2007-9-4 | Y | Sudden death |
| 108 | 2007-9-6 | Y | Heart failure |
| 109 | 2007-9-7 | N | Other reason |
| 110 | 2008-11-21 | Y | Uncertain |
| 111 | 2008-11-7 | N | Gastric carcinoma |
| 112 | 2008-12-18 | Y | Cancer-UK |
| 113 | 2008-12-18 | N | Pneumonia |
| 114 | 2008-12-25 | N | An accident |
| 115 | 2008-12-4 | Y | Heart failure |
| 116 | 2008-12-8 | N | Other reason |
| 117 | 2008-2-21 | Y | Uncertain |
| 118 | 2008-3-21 | Y | Uncertain |
| 119 | 2008-3-27 | Y | Myocardial infarction |
| 120 | 2008-4-16 | N | Multiple organ dysfunction syndrome |
| 121 | 2008-4-21 | Y | Myocardial infarction |
| 122 | 2008-4-3 | Y | Myocardial infarction |
| 123 | 2008-5-12 | N | Other reason |
| 124 | 2008-5-21 | N | Other reason |
| 125 | 2008-5-26 | N | Lung cancer |
| 126 | 2008-6-18 | Y | Uncertain |
| 127 | 2008-6-4 | Y | Heart failure |
| 128 | 2008-6-5 | N | Other reason |
| 129 | 2008-7-1 | Y | Uncertain |
| 130 | 2008-7-14 | Y | Myocardial infarction |
| 131 | 2008-7-22 | N | Pancreatic cancer |
| 132 | 2008-7-31 | N | Multiple organ dysfunction syndrome |
| 133 | 2008-8-21 | Y | Uncertain |
| 134 | 2008-8-8 | Y | Uncertain |
| 135 | 2008-9-1 | N | Lung cancer |
| 136 | 2008/9/25 | Y | Myocardial infarction |
